# Poison in the Nursery

**Published:** 1878-06

**Authors:** 


Poison in the Nursery.—The constant production of toilet novelties suggests a word of caution to those who purchase them. An analyst writes to the *Lancet* that on making a chemical analysis of a packet of violet powder he found, much to his astonishment, that it contained 25 per cent. of white arsenic.

In this instance the use of the powder had caused an epidemic among young children, many of whom died, the disease presenting every appearance of erysipelas. Here were mothers, while applying an innocent-looking toilet to their children, positively introducing into their systems a deadly and subtle poison.

Our readers may remember that during the trial in London of the notorious Madame Rachel, evidence was given that a violet powder sold by her contained a powerful irritant, which produced an unpleasant rash on the skin.

Comment upon these facts is unnecessary. Probably our readers will accept the hint to purchase such things in future from houses of undoubted respectability.—*The Medical Record.*

